# Supplementary material for: Predicting social media users’ indirect aggression through pre-trained models
Source: PeerJ Comput Sci. 2024 Sep 2;10:e2292. doi: 10.7717/peerj-cs.2292 (PMC11419620; doi:10.7717/peerj-cs.2292)
Supplement: Supplemental Information 1 [file peerj-cs-10-2292-s001.pdf]

English version	Chinese version
Please answer the questions below according to how you interact with your contacts on Weibo.<br>1 = Never behave this way,<br>2 = Very rarely behave this way,<br>3 = Rarely behave this way,<br>4 = Occasionally behave this way,<br>5 = Often behave this way,<br>6 = Very often behave this way,<br>7 = Always behave this way	***请根据您与您在微博上关注的人的互动情况， 回答以下问题。***<br>1=从没有这种表现<br>2=非常少有这种表现<br>3=很少有这种表现<br>4=偶尔有这种表现<br>5=经常有这种表现<br>6=频繁有这种表现<br>7=一直有这种表现
[Social Exclusion]	***[社交排斥]***
Withheld information from one or more people that the rest of the community is let in on	对某些人(或某个人)隐瞒信息，但将这些信息告知其他人
Purposefully left one or more people out of activities	故意将某些人(或某个人)排除在活动之外
Made others not talk to one or more people	使其他人不与某些人(或某个人)交谈
Excluded one or more people from the community	在集体中排斥某些人(或某个人)
Used private in-jokes to exclude one or more people	使用私人的内部笑话排挤某些人(或某个人)
Spread rumours about one or more people	散布某些人(或某个人)的谣言
Made one or more people feel that they don’t fit in	让某些人(或某个人)感到自己难以融入
Stopped talking to one or more people	停止与某些人(或某个人)交谈
Omitted one or more people from conversations on purpose	在谈话中故意忽略某些人(或某个人)
Turned the community against one or more people	让其他人反感某些人(或某个人)
[Malicious Humour]	***[恶意幽默]***
Used sarcasm to insult one or more people	用嘲讽的语气侮辱某些人(或某个人)
Made negative comments about one or more people’s physical appearance	对某些人(或某个人)的外貌作出负面评价
Imitated one or more people in front of others	在众人面前故意模仿某些人(或某个人)
Played a nasty practical joke on one or more people	对某些人(或某个人)开粗鲁的玩笑
Done something to try and make one or more people look stupid	做一些事让某些人(或某个人)出丑
Intentionally embarrassed one or more people around others	在大家面前故意让某些人(或某个人)难堪
Made fun of one or more people in public	在公众场合取笑某些人(或某个人)
Called one or more people names	给某些人(或某个人)起负面的外号
Criticised one or more people in public	在公众场合批评某些人(或某个人)
[Guilt Induction]	***[内疚诱导]***
Used my relationship with one or more people to try and get them to change a decision	利用我与某些人(或某个人)的关系来尝试促使他们改变决定

Tried to influence one or more people by making them feel guilty	试图通过让某些人(或某个人)产生内疚来影响他们
Used one or more people’s feelings to coerce them	利用某些人(或某个人)的感受来强迫他们
Used emotional blackmail on one or more people	对某些人(或某个人)使用情感勒索
Pretended to be hurt and/or angry with one or more people to make them feel bad about themselves	假装被某些人(或某个人)伤害和/或生气，使其为自己感到难过
Put undue pressure on one or more people	对某些人(或某个人)施加过分的压力
