# Supplementary material for: Predicting social media users’ indirect aggression through pre-trained models
Source: PeerJ Comput Sci. 2024 Sep 2;10:e2292. doi: 10.7717/peerj-cs.2292 (PMC11419620; doi:10.7717/peerj-cs.2292)
Supplement: Supplemental Information 2 [file peerj-cs-10-2292-s002.pdf]

Appendix A. Summary of direct aggression detection studies

Author	Method	Dataset	Aims	Context	Feature
Mazari et al., 2024	BERT + MLP	223 thousand comments from Wikipedia	Hate speech detection	Social media	Textual comments with categories' labels
Jaafar and Lachiri, 2023	MLP	An audiovisual dataset with a set of 21 videos	Aggression detection	Surveillance videos	Acoustic, visual, text, and extra-information
Alotaibi et al., 2021	CNN, BiRNN, and a transformer block	55 thousand tweets in Twitter	Comments' classification	Twitter	Textual comments with Tokenization
Sadiq et al., 2021	MLP	20,001 tweets with aggressive annotation	Aggression detection	Twitter	Textual tweets with Word Embedding
Balakrishnan et al., 2019	Random Forest	9 thousand tweets with labels	Cyberbullying classification	Twitter	Number of mentions, number of followers and following, popularity, favorite count, status count and number of hash tags
Risch and Krestel, 2018	MLP	15 thousand posts	Harmful posts' identification	Facebook	Textual posts with Word Embedding, Character N-grams, Word N-grams and Syntactic features
Chatzakou et al., 2017	Naive Bayes, Decision trees, Random Forest, and neural networks	1 million random tweets and 650 thousand tweets with 309 hate related hashtags	Detection of Cyberbullying and aggressive behavior	Twitter	Text, user information, and network
Al-Garadi et al., 2016	Naive Bayes, Random Forest, and KNN	2.5 million geo-tagged tweets	Cyberbullying detection	Twitter	Network, activity, user information, and content
Van Hee et al., 2015	Support Vector Machines	85 thousand Dutch posts	Cyberbullying classification	Ask.fm	Textual posts with bag-of-words features and sentiment lexicon features

REFERENCES

Mazari, A. C., Boudoukhani, N., and Djeffal, A. (2024). BERT-based ensemble learning for multi-aspect hate speech detection. *Cluster Computing*, 27(1): 325-339.

Jaafar, N., and Lachiri, Z. (2023). Multimodal fusion methods with deep neural networks and meta-information for aggression detection in surveillance. *Expert Systems with Applications*, 211: 118523.

Alotaibi, M., Alotaibi, B., and Razaque, A. (2021). A multichannel deep learning framework for cyberbullying detection on social media. *Electronics*, 10(21): 2664.

Sadiq, S., Mehmood, A., Ullah, S., Ahmad, M., Choi, G. S., and On, B. W. (2021). Aggression detection through deep neural model on twitter. *Future Generation Computer Systems*, 114: 120-129.

Balakrishnan, V., Khan, S., Fernandez, T., & Arabnia, H. R. (2019). Cyberbullying detection on twitter using Big Five and Dark Triad features. *Personality and individual differences,* 141: 252-257.

Risch, J., and Krestel, R. (2018). Aggression identification using deep learning and data augmentation. *In Proceedings of the first workshop on trolling, aggression and cyberbullying*, pp. 150-158.

Chatzakou, D., Kourtellis, N., Blackburn, J., De Cristofaro, E., Stringhini, G., and Vakali, A. (2017). Mean birds: Detecting aggression and bullying on twitter. *In Proceedings of the 2017 ACM on web science conference*, pp. 13-22.

Al-Garadi, M. A., Varathan, K. D., and Ravana, S. D. (2016). Cybercrime detection in online communications: The experimental case of cyberbullying detection in the Twitter network. *Computers in Human Behavior*, 63: 433-443.

Van Hee, C., Lefever, E., Verhoeven, B., Mennes, J., Desmet, B., De Pauw, G., ... and Hoste, V. (2015). Detection and fine-grained classification of cyberbullying events. *In Proceedings of the international conference recent advances in natural language processing*, pp. 672-680.
